# Agricultural Residues as Raw Materials for Pulp and Paper Production: Overview and Applications on Membrane Fabrication

**DOI:** 10.3390/membranes13020228

**Published:** 2023-02-14

**Authors:** Limenew Abate Worku, Archana Bachheti, Rakesh Kumar Bachheti, Cristiano E. Rodrigues Reis, Anuj Kumar Chandel

**Affiliations:** 1 Centre of Excellence in Nanotechnology, Addis Ababa Science and Technology University, Addis Ababa P.O. Box 16417, Ethiopia; 2 Department of Industrial Chemistry, Addis Ababa Science and Technology University, Addis Ababa P.O. Box 16417, Ethiopia; 3 Department of Environment Science, Graphic Era University, Dehradun 248002, India; 4 Guácimo Campus, EARTH University, Las Mercedes, Limón 4442-1000, Costa Rica; 5 Department of Biotechnology, Engineering School of Lorena (EEL), Estrada Municipal do Campinho, University of São Paulo (USP), Lorena 12602-810, São Paulo, Brazil

**Keywords:** pulp, paper, agricultural residues, pulping, non-woody biomass

## Abstract

The need for pulp and paper has risen significantly due to exponential population growth, industrialization, and urbanization. Most paper manufacturing industries use wood fibers to meet pulp and paper requirements. The shortage of fibrous wood resources and increased deforestation are linked to the excessive dependence on wood for pulp and paper production. Therefore, non-wood substitutes, including corn stalks, sugarcane bagasse, wheat, and rice straw, cotton stalks, and others, may greatly alleviate the shortage of raw materials used to make pulp and paper. Non-woody raw materials can be pulped easily using soda/soda-AQ (anthraquinone), organosolv, and bio-pulping. The use of agricultural residues can also play a pivotal role in the development of polymeric membranes separating different molecular weight cut-off molecules from a variety of feedstocks in industries. These membranes range in applications from water purification to medicinal uses. Considering that some farmers still burn agricultural residues on the fields, resulting in significant air pollution and health issues, the use of agricultural residues in paper manufacturing can eventually help these producers to get better financial outcomes from the grown crop. This paper reviews the current trends in the technological pitch of pulp and paper production from agricultural residues using different pulping methods, with an insight into the application of membranes developed from lignocellulosic materials.

## 1. Introduction to Agricultural Residues within the Pulp Industry

**Figure 1:**
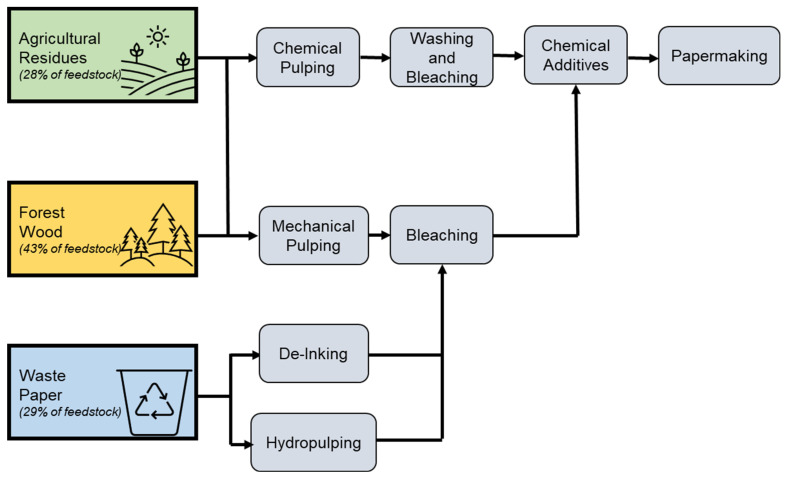
Types of raw material and their processing stages in papermaking with estimates from the Indian market in 2018 [4].

Pulp and paper mills are an essential part of the global economy and provide a wide variety of products for various uses. Because of their rapid industrial development and urbanization, the consumption of paper has constantly increased over the past decades in high-income countries, such as the United States, many in the European Union, and Japan [1]. Agricultural residues and weed plant residues can cater to the growing demand for paper in the packaging sector, as well as hygiene and sanitation products, such as paper towels, toilet paper, and disposable makeup wipes, among others [2,3]. In India, for instance, agricultural residues account for approximately 28% of the total feedstock used in the pulp and paper industry [4], supplied mainly from bagasse, wheat and rice straws, and cotton stalks. Figure 1 describes, overall, the types of raw material and their processing stages in paper formation. It can be seen from Figure 1 that, among the raw materials, forest wood remains the most common raw material. Nonetheless, the paper produced from either of three clusters remains as a fabric material, produced by removing water from a slurry containing cellulosic fibers. This process, in a nutshell, can be accomplished by the removal of lignin and, often, hemicellulose from the slurry, as described further in this article.

The use of forest resources to make pulp has increased in recent years, having a variety of industries, such as furniture manufacturing, construction, and others, using wood as a raw material. The pulp and paper industry relies primarily on the uses of fibrous wood. This overreliance on fibrous wood is linked to significant environmental problems, including deforestation, greenhouse gas emissions, and global warming [5]. Other raw material that also bear cellulosic fiber structures, such as non-woody plants, could therefore be considered for the manufacturing of pulp and paper to lessen the environmental problems associated with its use, [3] as non-woody plant source materials reduce the amount of wood used in the papermaking process [6], considering that around 60% of cellulose fiber is produced from non-wood materials, such as jute fibers, grasses, reed, bamboo, corn stover, sugarcane bagasse, and others. Some Asian markets, such as China and India, rely heavily on bagasse and wheat straw, both of which contribute to approximately 70% of the requirement to the pulp industry as raw materials [7]. These agricultural residues have, then, the potential to replace woody lignocellulosic biomass since they can be sustainable and affordable sources of cellulosic fiber. Their remnants are structurally similar to other plant fibers utilized in production, in terms of composition and physical and chemical characteristics [8]. Agricultural residues, like hemp, jute, kenaf, rice and wheat straw, and sugarcane bagasse, are promising pulp and paper-producing alternatives to woody materials [9].

Jiménez et al. [10] reported that straws with high fiber content present robust bleaching properties and are easily pulped, which makes them appropriate candidates to produce high-quality paper. Fibrous agricultural residues have the advantage, when compared to woody biomass, of being extensively scattered throughout the world. Additionally, plants used in the production of pulp and paper may be cultivated in unfavorable soil, and require little precipitation, offering a predictable feedstock for the industry. Non-wood pulp fibers have, in fact, been identified as an appropriate feedstock for paper production to alleviate deforestation problems [9]. Agricultural residues, such as hemp and sugarcane bagasse, present higher concentrations of cellulose and lower levels of recalcitrant lignin when compared to the average woody biomass [9], and also present higher annual growth rates when compared to trees [11]. Nonetheless, on a global scale, forest woods still account for over 40% of the main raw material for producing paper, while agricultural residues account for less than 30% [4].

The disadvantage of using agricultural residues is that, traditionally, their pulp mills run on smaller scales and they tend to be bulkier. Straw, like many other agricultural residues, usually presents high water content levels, reaching up to 70% on a weight basis, which needs to be dropped to levels around or below 25% to avoid microbial spoilage [12]. Drying straw fibers presents unique challenges; since its fibers are frequently shorter than those in softwoods, they typically drain more slowly. The high silica content of straw fibers, which makes the pulp recovery process challenging, is also one of the most significant issues with the chemical pulping of agricultural residues [13]. Chlorine-free bleaching techniques cannot produce agriculture residue pulps, with a high degree of brightness, due to their anatomical and chemical characteristics [14]. Several hundred chlorinated compounds were discovered in the bleaching waste liquors, which pose health dangers, including liver and kidney damage, hormone issues, and reduced gonad development in many living things found inside water bodies [15]. Soil and marine life fertility are seriously threatened by the polluted effluent discharges of bleached wastewater [15].

Environmentally friendly technologies, such as enzymatic treatments, offer low-cost alternatives for bleaching pulps. Xylanase is frequently employed in the pretreatment of pulp bleaching. It alleviates the drawbacks of agricultural residues by reducing the chlorine dioxide amount used for bleaching purposes [16] up to 20%, without affecting pulp quality. Plant pollution can be decreased through O_2_ delignification, totally chlorine free processes, elemental chlorine free technologies, oxidative alkaline extraction, replacement of Cl_2_ with ClO_2_, using different pretreatment, and improved pulp washing [17]. Extended delignification focuses on removing extra lignin before bleaching the pulp and dramatically lowering the chlorine concentration [18]. For example, the kappa number of rice straw can be reduced by up to 43% by delignification [19]. As a result, the chemical requirements of the bleaching sequence will be decreased, and the pollutant load will be reduced significantly [20].

## 2. Characteristics of Agricultural Residues and Their Pulping Process

Compared to wood fibers, non-woody materials tend to have a wider range of physicochemical properties [7]. They vary based on the species and regional factors, like soil and temperature [21]. Non-woody fiber-rich materials typically bear higher levels of hemicellulose, silica, and nutrients than wood [22]. The essential physical characteristics of non-wood raw materials are low bulk density, high fiber content, and short fiber length [22]. For instance, sugarcane bagasse, which is commonly used in the pulp and paper industry, presents about 45% of its dry matter as cellulose and 74% of holocellulose, with a similar fiber ratio when compared to hardwoods [23]. Besides bagasse, rye, kenaf, hemp, jute, and reed are also often used in the manufacturing of writing and printing paper [24]. Table 1 and Table 2 present, respectively, the fiber morphology of some widely used agricultural fiber-rich materials and the chemical characterization of these residues.

Pulping is breaking bonds between fibers, within raw materials, using chemical or mechanical means. Mechanical pulping, chemical pulping, and semi-chemical pulping are the most often utilized pulping processes [47]. Mechanical pulping works on separating fibers from each other using mechanical energy, and causes slow disruption of fiber connections, releasing single fibers, fiber bundles, and fiber fragments [48]. In this pulping procedure, the soaked fiber is pounded or ground with a grindstone to make the pulp [48]. Chemical pulping, on the other hand, involves adding chemicals that break down hemicelluloses and lignin into small water-soluble molecules, finally separating the fibers. After that, the molecules are rinsed away from the cellulose fibers, without depolymerizing them [49]. Chemical pulping methods are usually preferred due to their ability to leave the biomass fibers intact and increase the flexibility and conformability of the undried fibers [49]. The most used chemical procedures are organosolv pulping, soda pulping, sulfite pulping, and kraft pulping.

A commonly used method for lignocellulosic biomass fractionation is organosolv delignification [50]. Organosolv delignification has been proposed as an environmentally friendly and alternative pulp production method. In industrial paper-making processes, organosolv is a pulping technique that uses an organic solvent to solubilize lignin and hemicellulose. It has been considered in the context of both pulp and paper manufacture, and biorefining, for the subsequent conversion of cellulose to fuel ethanol. Various organic solvents have been proposed for pulping, including organic acids, ketones, glycols, alcohols, and esters [51]. Distillation makes it simple to recover low-boiling solvents, like acetone, ethanol, and methanol, whereas the organosolv techniques use high-boiling solvents, like ethanolamine and ethylene glycol, that may be used at low pressure [51]. This method can generate pulps with high yield, high strength, excellent brightness, and low residual lignin content [52]. Organic solvents are useful for producing lignin-based adhesives and can be easily recovered at high purity, with low kappa numbers and low environmental impacts. Ref. [53] employed organosolv pulping to remove hemicelluloses and lignin from date palm fronds in the United Arab Emirates. Ref. [54] used organosolv pulping to extract non-cellulosic material from rice straw. According to this study, cellulose from rice straw can be used as a substitute for sheets. In fact, organosolv procedures are used to pretreat the rapeseed straw for pulp and paper making [55,56].

In the pulp and paper industry, the soda process is one of the most common ways to produce chemical pulp from agricultural fibrous residues. The soda pulping process involves cooking the biomass with sodium carbonate (6–8% solution), or a mixture of sodium carbonate (50–85% solution) and sodium hydroxide (15–50 percent solution), usually at high steam pressures and temperatures, for a short time. Jimenez et al. [8] described a pulping process for abaca fibers using 1100 kPa of steam pressure, for 14 min, at a temperature range of 150–160 °C. The soda process is often reported to be advantageous when compared to other methods, given it provides feasible opportunities for chemical recovery and a reduced cooking time. However, given how the process is designed, the yields of recovered pulping chemicals can be low.

This pulping method also has the advantage of producing sulfate-free pulp, and pulping additives can enhance the paper’s quality and strength. Since it moves electrons from the biomass carbohydrates to its intermediate structures during the lignin’s degradation, anthraquinone is considered an appropriate additive [57]. During alkaline pulping, anthraquinone works as a redox catalyst, leading to a carboxylic acid being formed by reducing aldehyde end groups of carbohydrates, and inhibiting alkaline de-polymerization, resulting in increased yield, decreased solid formation in the black liquor solution, and a lower kappa number. While this process leads to lower pulp brightness after bleaching, the soda-anthraquinone pulping operation is known to save considerable energy [58]. Soda-adapted processes have been applied to fibers from canola stalks, rapeseed straw, and sunflower stalks [59], attaining yields in the range of 30–40%. Saeed et al. [60] investigated the paper qualities produced from agricultural residues, such as sesame, karkadeh, and okra stalks utilizing soda, and soda-anthraquinone pulping. This study demonstrated a decreased kappa number, increased pulp production, and enhanced paper properties. Soda-anthraquinone processes can be complemented by other additives, such as monoethanolamine [61], which is known to improve the pulping process of recalcitrant fibers. Okra stalks were also reported as feedstock for a soda-anthraquinone pulping process [62], in which the authors reported a maximum yield of 32%. These lower yields, with reported good papermaking characteristics [62], could be blended with other pulps to lead to higher yield processes. A life cycle assessment of hemp and flax indicated that most of the activities inside the pulp mill present minor contributions to the majority of the impact categories [63].

Kraft pulping is another well-known alkaline process; it is used in the chemical pulping method to make fibers of cellulose from raw wood and other sources of the plant. Essentially, an alkaline solution (such as sodium hydroxide or sodium sulfide) is deployed in the process, and lignin depolymerization occurs primarily in ether linkages connected to the position or monolignols [64]. The polymer of lignin is broken into small chains by breaking the ether bond and dissolving in an alkaline solution to form a lignin solution system, known as black liquor. At high temperatures, white liquor, for instance, containing sodium sulfide (Na_2_S) and NaOH, is added to the chipped material, and cellulose is recovered by dissolving almost all the lignin and a large part of the hemicelluloses into the black liquor. Lignin is broken down into small molecules during this process by the cleavage of ether linkages (mostly -O-4 bonds). Kraft processes have been suggested to operate with blends of hardwood and straw and corn stover, since these agricultural residues enhance the xylan on the mechanical properties of pulp [65]. This has been corroborated by Tavast et al. [66]. Given the fact that most agricultural residues have lower lignin content, the kraft cooking process may not be the most appropriate for such materials. Nonetheless, the co-cooking of these with hardwoods is promising.

Sulfite pulping is an acid pulping process that is typically carried out in a pH range of 1.5 to 5. In this process, sulfuric acid is used to digest wood chips, at a temperature range of 120 °C to 180 °C, for 1–5 h. Because the sulfur content is high in sulfonated lignin, around 5 to 6% of the lignin obtained by the process of sulfite pulping is used as fuel for the pulp mill and to recover inorganic components [67]. Unlike other chemical pulping processes, it does not involve the cleavage of β-O-4 linkages, but α-O-4 linkages. Furthermore, the occurrence of condensation events through the reaction of the intermediate carbocation, with an electron-rich carbon atom, is an important step in the preparation of a high dispersity index and lignosulfonates [64].

Some methods involve the combination of two or more of the aforementioned approaches. Semi-chemical pulping processes, for instance, involve chemical softening and refining the pulp in a mechanical pulper, using a lower quantity of chemicals. Chemi-mechanical pulping has several advantages, including a higher yield and less chemical use, which benefit the environment. The pulps produced through this process tend to lead to higher yields, owing to the use of fewer chemicals. Gonzalo et al. [68] experimented with six different agricultural residues to find new starting materials for the semi-chemical soda pulping process, such as broad bean, okra, stems of bell chili peppers, asparagus, and peas. Except for pea stems, the results revealed strong pulping yields of more than 60%. Studies in this area have resulted in identifying appropriate procedures and developing innovative systems that result in increased yield and pulp quality. Salehi [69] investigated high-yield chemi-mechanical pulping of bagasse at 95 °C, using 10, 15, and 20% sodium hydroxide, and 10, 15, and 20 min cooking intervals. The findings of the experiments reveal that durian rinds have a lot of potential for the industry as a non-wood-based raw material [70].

In biological retreatment, microorganisms and their enzymatic complex are employed to break down lignin and modify lignocellulosic structures. Because they can mineralize lignin to CO_2_ and water in pure culture, white-rot fungi are often reported as the most appropriate candidates for lignin decomposition [71]. Several white-rot fungi, including *Phanerochaete chrysosporium*, *Ceriporiopsis subvermispora*, *Phlebia subserialis*, and *Pleurotus ostreatus*, may successfully metabolize lignin in a wide range of lignocellulosic substrates [72]. Because it is neither poisonous nor chemically harmful, the biological pulping process can be carried out at room temperature. Furthermore, the process consumes less energy in subsequent stages [73]. The ability of enzyme extracts, derived from *Polyporus fomentarius*, *Bjerkandera adusta*, and *Trametes versicolor*, to promote the breakdown of cell wall components of wheat straw was evaluated in vitro using pineapple leaf fiber as a raw material [7]. According to the findings, bioprocessing may also induce enzymes to attack lignocellulosic substrates, limiting excessive polysaccharide consumption. *Trametes versicolor* and *P. ostreatus* were used to bio-pulp. According to the data, pulp yield was 35% to 50% with *T. versicolor*, and 55% to 70% with *P. ostreatus.* Longer colonization durations resulted in lower levels of lignin and extractives, and higher levels of holocellulose [74]. In investigating straws of wheat as a natural substrate, seven fungal strains were investigated independently, and in various combinations, to know their lignin-degrading capacities [75]. When investigated individually, the fungus *P. chrysosporium* produced the greatest loss of total organic matter (26.45%) and lignin component (28.93%). The relationships between various types of fungi revealed that ligninolysis was improved to varying degrees in particular combinations of white-rot plus brown-rot, white-rot plus white-rot, and white-rot plus soft-rot fungi. The combination of *Deadaleaflavida* and *P. chrysosporium* produced the best lignin loss of 36.27% [75].

Pulping is affected by the nature of the fiber and the quality of the chips formed from it, as well as by alkali charge, sulfide, temperature, kappa number, and pulping equipment [76]. Chemical properties (holocellulose, alpha-cellulose, and lignin), fiber morphology (well thickness, fiber length, fiber width, Runkel ratio, flexibility coefficient), and fiber quality, such as coarseness, external fibrillation, kink indexes, curl and pulp fines, all influence pulp and papermaking parameters. Kappa number, tensile index, pulp yield, tear index, and burst index also affect the quality of non-woody pulp [76].

Chemical properties of the wood are primarily considered, in the pulping process, to assess the quality of the fibrous raw material. The cellulose, hemicellulose, and lignin that make up the cell wall are the major or structural constituents of the plant cell, while the extractives and inorganic compounds are the secondary or non-structural ingredients. The chemical makeup of the wood that will be used to make pulp is very important since it directly affects how the pulping process works. For example, Gomide et al. [77] showed the importance of lignin and extractives content in wood, such as quality standards from eucalyptus to cellulose production, because they have a big impact on the kraft pulping process and have good correlations with it. Similarly, in agricultural residues, the higher the lignin and extractive content in the wood, the more reagents are used, and the lower the pulping yield. Jute fiber produced the most pulp (63.97%), in a different study, when compared to other fibers like rice straw, wheat straw, okra stalks, maize stalks, and cotton stalks. This is because jute fiber has the lowest levels of Klason lignin (14.6%) and the highest levels of holocellulose (71.9%) and alpha-cellulose (54.3%). This demonstrates a favorable correlation between holocellulose production and pulp production.

One of the most crucial variables assessed in chemical pulping is the kappa number. It measures the amount of 0.1 N potassium permanganate solution absorbed by 1 g of moisture-free pulp in an acidic medium, expressed in milliliters (mL), to show the degree of pulp delignification [78]. Kappa’s first immediate effect is on pulping yield. Delignification degree and yield are two factors that are inversely connected; for example, lowering kappa and raising the pulp delignification degree both reduce pulping yields [79]. Conversely, a low level of delignification (greater kappa) results in higher yields [80], which has a favorable effect on the ability of an industrial plant to produce good-quality pulps.

Table 3 categorically summarizes the various pulping technologies, applied to the various agricultural residues, for the production of pulp and paper.

## 3. Bleaching of Fibrous Agricultural Residues

**Figure 2:**
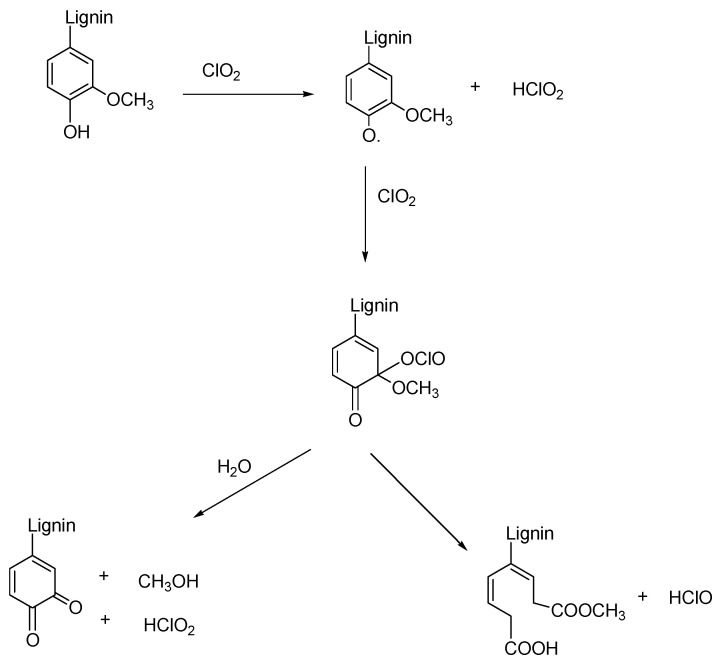
Reaction series of bleaching using ClO_2_ [82].

Pulp bleaching aims to improve the pulp’s brightness by eliminating or altering colored components. Color is principally controlled by the chromophoric groups of lignin [82]. Bleaching is considered a continuation of the pulping process because it removes residual lignin; however, it is done in stages to be gentler and less destructive [83]. Due to worldwide trends and an increased need for more environmentally friendly bleaching methods, elemental chlorine free and total chlorine free are being utilized more frequently to reduce the severity of chlorinated organic compounds [84]. The difference between the two processes is the usage of chlorine dioxide, which is employed in elemental chlorine free, but not in total chlorine free. The pulp and paper industries have greatly decreased the production and release of chlorinated organic material, which are polluted substances, into the aquatic environment by using chlorine dioxide (ClO_2_) as a substitute for elemental chlorine (Cl_2_) [85]. In comparison to conventional chlorine-based bleaching and pulping, oxygen-containing oxidative chemicals, like hydrogen peroxide, ozone, and molecular oxygen, have emerged as one of the most significant, affordable, and environmentally friendly substitutes [86]. Hydrogen peroxide (H_2_O_2_) is also commonly referred to as an effective way to bleach wastepaper pulp since it works well at increasing pulp brightness and complies with environmental laws. Hydrogen peroxide reacts with chromophores in alkaline conditions, changing their composition to colorless forms. The reaction series in Figure 2 demonstrates the ring opening of lignin residues, which renders a less intense brown color in the pulp. The mechanism of all the other bleaching processes follows the same principle of rupturing chromophoric structures.

In biological-based bleaching, the pulp is bleached using enzymes and microorganisms. It is based on the employment of primarily fungal cells or enzymes, while some bacteria are also known to promote direct depolymerization of lignin. The use of oxidative enzymes, produced by these microorganisms, in developing chlorine-free pulp bleaching methods has been a topic of research for decades. Today, enzymatic bleaching utilizing xylanases is being investigated as a straightforward and practical substitute for the manufacture of cleaner pulp [87]. Although biological bleaching tends to generate pulp with lower brightness when compared to chemical methods, it can be a cost-efficient method, effective, and it also lessens the contamination caused by chemical bleaching [88]. Hemicellulolytic enzymes, particularly xylanases, are commercially utilized for pulp bleaching [89]. Many industrial xylanases have also exhibited features incompatible with pulp bleaching [90]. Through a series of steps that include chlorine dioxide, alkali, chlorine dioxide, optional hypochlorite, and elemental chlorine, the bleaching process eliminates between 5 and 10% of the remaining lignin [91]. For instance, laccase and lignin peroxidase are two oxidative enzymes produced by white-rot fungi, and are frequently used for paper and pulp production [92].

Peroxide is used to bleach pulps that have formed due to the mechanical process, which has about the same proportion of lignin as the original raw material. Chemical and semi-chemical pulps are often bleached in one or more phases with hypochlorites, or directly with chlorine compounds. Bleaching of chemical pulps often involves several stages. Typically, the first stage is the application of chlorine, followed by an alkali wash and hypochlorite in the third step [15]. Pulps made from agricultural waste material, however, require less amounts of chlorine than wood pulps [81].

## 4. Application to the Development of Membranes and Their Use

As previously disclosed, agricultural residues, such as straw and bagasse, have been increasingly recognized as potential raw materials for the production of pulp and paper. Furthermore, the development of advanced processing techniques has led to the development of new and innovative applications, such as the creation of high-performance membranes. The fibers extracted and modified during the pulping of agricultural fibrous residues can be transformed into membranes through various methods, such as casting, solution-diffusion, and phase-inversion. These materials are often considered promising alternatives for replacing and enhancing operations that are energy-intensive across many industrial sectors.

The efficiency of membrane separation is largely dependent on the choice of material used in membrane fabrication, which affects permeability, membrane structure and thickness, and module design [93]. One type of membrane material that has received considerable attention is the mixed matrix membranes (MMM), which are composite membranes obtained by integrating a filler material into a continuous polymer matrix [94]. The filler can be either organic or inorganic, and the goal is to take advantage of the micropores in the inorganic filler for improved interaction with the target adsorbate [95]. By incorporating an organic or inorganic filler, the synergistic properties of both materials can be applied. Solving the trade-off of polymeric membranes can be achieved through the use of mixed matrix or composite membranes. In a lignocellulosic biomass-based MMM hybrid system, for instance, the targeted molecules are sorbed by the membrane and diffuse through it [96]. The thermodynamic and kinetic parameters control this mechanism and have already been shown to be beneficial in gas separations and other applications.

MMM systems with cellulose extracted from natural sources, such as agro-based materials, have been created by blending it with polymers, carbon, and other materials [97]. The polymer acts as the continuous phase, while the cellulose functions as the dispersed phase, which can result in a defective morphology if the polymer matrix becomes rigidified. Mixed matrix membranes with cellulose show excellent mechanical strength and are utilized in various industries, including gas separation, water purification, tissue engineering, and food packaging [97]. Researchers are continually exploring ways to modify polymers and cellulose for environmental and separation benefits by reducing its crystallinity, decreasing particle size to the nanoscale, increasing tensile strength, and accessing its hydroxyl groups [98]. Various functional groups have been used to modify polymers, including amine, aniline, methacrylate, polyvinyl alcohol, and polyethylene oxide [99].

Lignocellulosic materials have been considered potential alternative sources for the fabrication of membranes. This approach, however, is linked to significant challenges, which, when overcome, may lead to groundbreaking changes in well-established separation processes. The use of lignocellulose-based membranes is affected by the complexity of the structure and by the low solubility of cellulose in aqueous and organic compounds. This characteristic makes them a particularly interesting candidate for the fabrication of membranes applied in harsh environments, such as those needed in the preparation of organic solvent nanofiltration [100]. Mohamed et al. [101] extracted cellulose nanocrystals from kapok fiber, a cellulosic source, and subsequently created self-assembled cellulose nanocrystal membranes, using these nanocrystals. Through a water suspension casting and evaporation procedure, the cellulose fibers derived from the alkali extraction and acid hydrolysis of kapok fiber were combined into a nanoporous membrane. Cellulose obtained from a non-dyed cotton bed sheet and dissolved in the mixture of (Emim) (OAc) and DMSO was directly used for UF membrane fabrication, without pre-treatment [102]. Cellulose concentration in the range of 5–7 wt.% was required to obtain membranes with better adhesion stability and uniform performance. By revalorizing its cellulosic component, Saad et al. [103] evaluated *Ziziphus lotus’s* potential for manufacturing carboxymethyl cellulose sorption membranes, with the capacity to adsorb methyl green from wastewaters. The outcome demonstrated the membrane’s potential for removing harmful dyes from wastewater. It was discovered that an adsorption membrane made from carboxymethylcellulose, obtained from *Ziziphus lotus*, was capable of removing 99% of the wastewater’s methyl green dye. To create biodegradable nanofiltration membranes, Alammar et al. [100] solubilized date seed biomass using ionic liquids and dimethyl sulfoxide. The developed membranes showed exceptional performance for oil-in-water separation and organic solvent nanofiltration.

Nanocellulose and its derivatives have been reported to be applied in the removal of dyes, heavy metals, aflatoxins, and fluoride from industrial wastewater [104]. These membranes are usually composed of cellulose and another compound to enhance their properties. For instance, the cellulose-CeO_2_ nanocomposite membrane, fabricated by Yao et al. [105], was based on the growth of CeO_2_ on a cellulose membrane. These nanofibers can accommodate a wide variety of oxides and metals in their structure, such as Pb, Ni, Au, Ag, TiO_2_, and SiO_2_, leading to a somehow tunable membrane. These membranes can also go through quaternization, i.e., the process behind crosslinking quaternary ammonium via covalent bonding with the reactive hydroxyl groups of the cellulose structures. These quaternary-ammonium membranes can be efficiently applied to the removal of anions from wastewater. Some further applications of biomass-derived membranes, that are not directly related to its separation processes, include the production of cellulose-nanofibril membranes for the fabrication of supercapacitor separators [106], electric heating systems [107], or their application in vivo in mesenchymal stem cells [108]. Sustainable nanocellulose has great potential for use in a variety of energy-related applications. In order to simultaneously increase the mechanical robustness, water-stability, and proton conductivity (by inserting -SO_3_H^+^ functional groups), Selyanchyn et al. (2022) [109] constructed proton exchange membranes (PEMs) using nanocrystalline cellulose. The combination of crosslinking and sulfonation has been previously applied to simultaneously improve proton conductivity in polymers, and membrane integrity or aqueous stability.

The properties of high specific surface and non-toxicity make cellulose-derived membranes possible suitable candidates to be used in water purification. Mahfoudhi and Boufi [110] reviewed some applications of nanocellulose for environmental remediation and concluded that the functionalization of the hydroxyl groups of the cellulose structure extends the possibility of adsorption of a wide variety of pollutants present in wastewater. Snyder et al. [111] developed a membrane from eucalyptus-derived nanofibers, which was doped with TiO_2_, Ag, and Au to remove methylene blue from wastewater. Stephen et al. [112] used cellulose nanofibrils, modified with oxolane-2,5-dione, to act as adsorbents for Pb from wastewater. The modification of the hydroxyl groups can be achieved by a number of methods, of which oxidation and functionalization stand as, potentially, the most common methods in the literature. The oxidation of the hydroxyl groups in cellulose leads to carboxylated cellulose, which is denser in charge, which has been proven to adsorb a multitude of ions, including radioactive metals [113,114].

Nanocellulose-based membranes have been found to be effective in pressure-driven filtration processes, such as micro-, ultra-, and nanofiltration [97,115]. They can also be used in concentration-driven forward osmosis and thermally-driven membrane distillation, potentially reducing energy consumption and making water purification accessible in rural areas using solar power [8]. The use of cellulose in the fabrication of biomass-derived membranes is, thus, a promising alternative to petroleum-based resins and other plastics that are commonly used in these materials. These examples illustrate some of the most critical applications of lignocellulosic-biomass-derived membranes. However, the number of processes that can benefit from cellulose-based membranes is significantly greater and can be seen in a plethora of industrial applications [96]. From a broader perspective, one must consider the assessment of the life cycle of cellulose products as a crucial step because of the growing focus on developing sustainable and renewable nanocellulose. However, there is a lack of comprehensive data on the impacts of cellulose products on different environmental factors, such as human health, air emissions, and waste disposal, leading to inadequate evaluations. Additionally, most laboratory processes do not consider the end-of-life stage of cellulose materials, making it difficult to scale up production.

To overcome these challenges, it is important to have a thorough understanding of lignification mechanisms and to use appropriate pretreatment techniques for lignocellulosic biomass to make it more durable and to prevent fouling. Collaboration between material chemists, polymer scientists, and chemical and material engineers is needed to develop next-generation membrane materials that can withstand physical aging and plasticization, making them suitable for industrial use. Extracting cellulose and hemicellulose from various sources, like straw and grass, is a common challenge, which can be addressed by carefully selecting an appropriate pretreatment technique for high purity and yield. Reducing the particle size to the nanoscale and carefully examining the reactivity of group characteristics of lignocellulosic biomass will improve its performance and compatibility during incorporation into the polymer matrix. In this sense, while the links between the pulping process of agricultural residues and nanocellulose to be used in membrane fabrication exist, they are fairly unexplored by the scientific community.

## 5. Conclusions and Prospects

High dependency on wood for the production of pulp and paper production creates major environmental problems, like global warming, greenhouse gas emissions, deforestation, and dwindling fibrous wood resources. As a result, alternative sustainable raw material sources for pulp and paper production might be researched to address environmental concerns. Non-wood fibers, such as agricultural residues, can be used to make pulp and paper more sustainably. Agricultural residues could be suitable for cellulosic fiber sources in the pulp and paper industry since they are renewable, inexpensive, and abundant. Cotton stalks, rice straws, wheat straws, sugarcane bagasse, and corn stalks are all non-wood plant fibers currently employed as papermaking raw materials by many paper manufacturing companies. High silica content harms paper machines’ efficacy and mills’ chemical recovery. Silica remaining in the pulp, and the pulp’s drainage, can be hampered by its short fiber length. This challenge necessitates the development of appropriate technology to address it on the shop floor. In developing countries, farmers commonly burn agricultural byproducts in the field, due to challenges with residual management, resulting in significant air pollution and accompanying health issues. Therefore, this review paper could bring awareness to the farmers using agricultural residues for the production of pulp and paper. Traditional pulp bleaching technologies involving chlorine and chlorine compounds are still used, putting an enormous burden on environmental resources. Thus, less harmful cooking and pulp bleaching techniques must be used to convert agricultural residues into pulp and paper. Furthermore, the developed products can be converted to specialized membranes, which can be applied to a wide range of industries, ranging from water purification to medicinal uses. To properly address the environmental issue, new and faster-growing technology with less chemical and water footprints must be developed.

## Figures and Tables

**Table 1:** Fiber characteristics of some important agricultural residues.

Agriculture Residue	Wall Thickness (µm)	Lumen Diameter (µm)	Fiber Width (µm)	Fiber Length (mm)	Reference
Sugarcane bagasse	5.63	9.72	20.96	1.59	[25]
Banana fiber	5.20	29.40	41.60	2.84	[26]
Barley straw	4.07	6.97	15.26	0.67	[27]
Canola straw	7.43	11.9	28	1.21	[28]
Coconut husk/coir fibers	2.91–4.02	10.71–12.97	17.52–20.68	0.69–1.06	[29]
Coconut husk/coir fibers	4.41	13.59	20.09	0.84	[30]
Coconut husk/coir fibers	3.30	10.71	17.60	0.67	[27]
Sunflower stalk	5.85	11.12	22.84	0.96	[31]
Sunflower stalk	5.90	11.90	23.70	0.96	[32]
Kenaf bark	4.2	11.9	21.9	2.32	[33]
Kenaf fiber (core)	4.3	13.2	22.2	0.74	[34]
Maize stalks	2.00	4.40	8.40	1.52	[35]
Maize stalks	4.19	10.92	20.12	0.88	[31]
Maize stalks	8.82	13.67	30.19	1.52	[36]
Maize Husk	8.82	13.67	30.19	1.37	[36]
Maize straw	3.68	9.73	17.18	0.75	[27]
Miscanthus x giganteus Stalks	4.64	5.76	15.17	0.50	[27]
Rapeseed straw	1.17	23.02	12.50	5.26	[37]
Rapeseed straw	4.31	15.50	24.12	0.95	[31]
Rapeseed straw	2.25	8.60	13.10	1.20	[38]
Rapeseed straw	4.91	19.10	28.00	1.03	[3]
Rapeseed up	4.25	9.73	18.36	0.71	[27]
Rapeseed low	3.86	11.04	18.99	0.57	[27]
Rice straw	3.02	4.52	10.77	0.54	[27]
Rice straw	1.50	1.90	4.90	0.66	[35]
Rice straw	3.16	4.57	10.89	0.83	[31]
Rice straw	1.83	5.6	11.6	0.78	[39]
Sugarcane	NA	NA	19.35–20.96	1.22–1.59	[40]
Sugarcane bagasse	4.50	10.25	19.86	1.15	[27]
Sugarcane bagasse	1.60	5.20	8.40	1.70	[41]
Sugarcane bagasse	7.74	6.27	21.40	1.51	[42]
Sugarcane bagasse	5.64	9.72	20.96	1.59	[40]
Sunflower stalk	4.80	13.23	22.99	0.64	[27]
Wheat straw	4.59	4.02	13.20	0.74	[43]
Wheat straw	4.13	8.34	16.87	0.78	[27]
Wheat straw	1.60	6.80	9.90	0.85	[35]

NA: not available. The variation in sugarcane bagasse fiber characteristics depends on multiple factors, e.g., climate, cultivation conditions, varieties, methodology, and geographical distribution.

**Table 2:** Proximate chemical analysis of some important agricultural residues.

Agriculture Residue	Holoce-Llulose (%)	Klason Lignin (%)	Hot Water Solubility (%)	Cold Water Solubility (%)	Alcohol Benzene Solubility (%)	1% NaOH Solubility (%)	Ash (%)	Reference
Barley straw	66.01	19.47	16.25	11.01	8.71	56.25	10.97	[44]
Cornstalk	69.92	18.16	16.82	14.64	8.57	46.43	7.75	[44]
Cotton stalk	62.79	23.79	17.91	15.05	8.36	48.88	4.99	[44]
Hemp	86.08	6.42	5.85	5.29	1.59	20.04	3.62	[44]
Cornstalk	82.10	7.30	12.60	NA	NA	69.60	24.9	[45]
Empty fruit bunches	80.00	17.00	3.50	NA	2.30	NA	3.5	[46]
Oil palm fronds	83.00	21.00	4.50	NA	2.30	NA	2.5	[46]
Oil palm trunks	73.00	25.00	5.50	NA	1.30	NA	2.5	[46]
Reed stalk	78.85	22.79	9.80	7.61	3.26	36.81	4.17	[44]
Rice straw	60.70	12–16	7.30	9.66	0.60	57.7	15–20	[44]
Rye straw	76.95	17.25	15.72	11.95	7.44	44.35	4.01	[44]
Sunflower stalk	66.85	14.43	24.26	21.08	7.48	50.05	7.99	[44]
Tobacco stalk	64.29	15.15	21.56	17.15	8.06	50.57	14.44	[44]
Wheat straw	69.84	22.33	14.71	11.33	9.33	53.67	11.63	[44]

**Table 3:** Comparative account of different pulping technologies applied to the various agro-residues. Adapted from [81].

Pulping Technology	Conditions (Agricultural Residues and Pulping Conditions)	Specific Features (Removal of Lignin, and Other Parameters)	Environmental Impact *
Thermo-mechanical process	Used for all agricultural residues	Softening of biomass by steam followed by fibrilization	*+++*
The whole wood fiber manufacturing process	Agricultural residues can be used for this process by using different mechanical means after steaming	Conversion of biomass tissue into the fibrous state without chemical action	++
Semi-chemical pulping process	Wheat straw, rice straw, corn stalks, cotton stalks	High yield of pulp with significant removal of lignin and hemicellulose	+++
Chemical pulping	Suitable for agricultural residues	Efficient delignification, the minimum requirement of fibrillization	++++
Sulfite process	The acid sulfite process is not suitable for pulping agricultural wastes	Using a buffered acid solution of calcium bisulfite or magnesium bisulfite. Conversion of lignin into soluble lignosulfonate acid and consequently lignosulfonates production	++++
Soda and modifications (e.g., soda-anthroquinone)	Silica is easily dissolved in an alkaline medium. Recommended for agricultural residues.	NaOH solution reacts with the free –OH groups in the lignin molecules and converts it into sodium ligninate (alcoholate)	++
Kraft process	Suitable for woody biomass. It produces stronger pulp than most other chemical and mechanical processes.	NaOH is replaced by Na_2_S enhancing the delignification	++++
Organosolv	Delignify bagasse, cotton stalks and wheat straw.	Using a broad range of organic solvents	++++
Bio-pulping	Use of microorganisms or enzymes in the pulping process	Enzymes from wood degrading fungi for selective degradation of lignin, low temperature and atmospheric pressure but requires long processing time	+

* +: environmentally friendly approach, ++: low impact, +++: medium impact, ++++: severe impact.

## Data Availability

Not applicable.
